# Osteochondroma of the Mandibular Condyle: A Rare Case Presentation With Literature Review

**DOI:** 10.7759/cureus.50355

**Published:** 2023-12-11

**Authors:** Dilasha Dhungel, Varun Rastogi, Nisha Maddheshiya, Sandhya Chaurasia, Karthikeyan Ramalingam

**Affiliations:** 1 Department of Oral Pathology, Universal College of Medical Sciences and Teaching Hospital, Bhairahawa, NPL; 2 Department of Oral Medicine and Radiology, Faculty of Medical Sciences, Institute of Medical Sciences, Banaras Hindu University, Varanasi, IND; 3 Department of Oral Pathology and Microbiology, Saveetha Dental College and Hospitals, Saveetha Institute of Medical and Technical Sciences, Saveetha University, Chennai, IND

**Keywords:** posterior crossbite, excision, mandible, computed tomography, ct, deviation, condylectomy, histopathology, mandibular condyle, osteochondroma

## Abstract

Osteochondromas (OCs) are benign bone tumors characterized by their growth with a cartilage cap and typically occurring at the ends of long bones. Their occurrence in the head and neck region is infrequent, accounting for only around 1% of head and neck tumors. Notably, the mandibular coronoid process and the mandibular condyle are the primary sites where an OC is reported. Patients often exhibit facial asymmetry, limited mouth opening, and malocclusion. Possible treatment options depending on the condition include partial or total condylectomy, vertical ramus osteotomy, and supplementary orthognathic surgery. The recurrence rate of under 1%- 2% is reported after local resection.

In this case report, we present a unique case of an OC in a 27-year-old woman. It involved the mandibular condyle, resulting in a left-sided mouth deviation while opening and closing her mouth. The purpose of this article is to detail the clinical and radiographic features, histopathological aspects, and treatment strategies and differentiate potential diagnoses, for such OCs.

## Introduction

An osteochondroma (OC), also referred to as osteocartilaginous exostosis, is a benign neoplasm within the skeletal system [1,2]. Typically manifesting in endochondral bones, this condition is often devoid of symptoms and presents as a protruding growth on the bone surfaces [3]. Approximately 85% of OCs occur as single lesions (pedunculated or sessile types), while the remaining 15% are multiple OCs, often as hereditary disorders associated with multiple exostoses (HME) [4,5].

There are approximately 35.8% of benign bone tumors, with a reported 2% recurrence rate [1,6,7]. The OC is a rarity in the head and neck region. It has been observed in diverse locations such as the skull base, maxillary sinus, zygomatic arch, and mandible [2,8-11]. The incidence in the craniofacial region is 0.6%. The mean patient age is 39.7 years and the peak age range is in the fourth decade. Female incidence is reported to be greater than males [9]. There are a total of 98 cases of mandibular condylar OCs reported between 1927 and 2010 [11]. The OC has been reported to involve the mandibular condyle and coronoid process. Bilateral condylar involvement has also been noted [8,10]. The OC involving the condyle has been linked to difficulties in mouth opening, dental malocclusion, and facial asymmetry [2,3,11].

In this report, we showcase a scenario involving a 27-year-old woman diagnosed with an OC located on the mandibular condyle. We present the clinical and histopathological observations, accompanied by an exploration of existing literature on the subject.

## Case presentation

**Figure 1:**
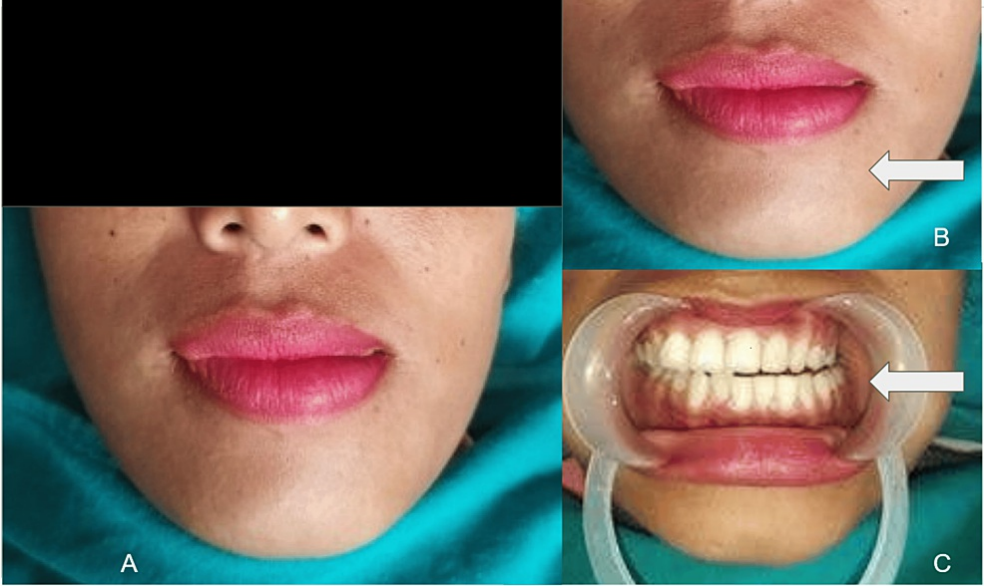
Clinical pictures Clinical picture showing the extra-oral deviation to the left side (A, B) and intra-oral view of the open bite with crossbite on the left side (C)

A 27-year-old woman presented herself at the outpatient department with a concern about her limited ability to open her mouth over the previous 12 months. Past medical, surgical, and dental history was non-contributory. Clinical examination showed a left-sided deviation of the mouth while opening and closing the mandible. Her mouth opening was around 18mm measured using a graduated scale. Extra-oral palpation in the temporomandibular joint region showed restricted movement (Figures 1A, 1B). The intra-oral view showed a crossbite on the left side (Figure 1C).

**Figure 2:**
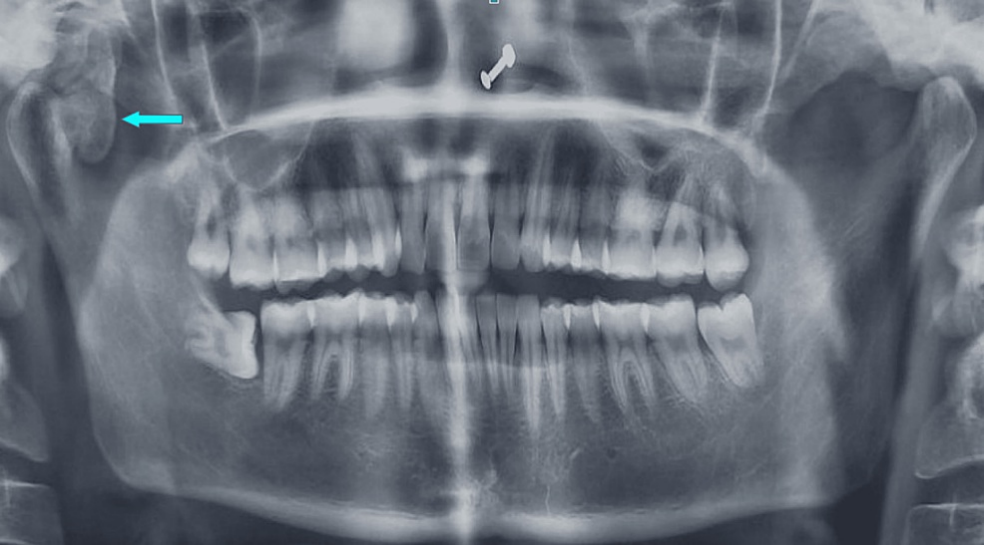
Orthopantomogram Orthopantomogram showing the lesion involving the right mandibular condyle

The orthopantomogram revealed a single well-defined radiopaque bony overgrowth seen in the anterior aspect of the right condyle. A well-defined radiopaque structure is visible on the anteromedial surface of the right condyle, extending inferiorly to the neck of the condyle and superiorly to the intra-articular space measuring approximately 2 x 3 cm in size (Figure 2).

**Figure 3:**
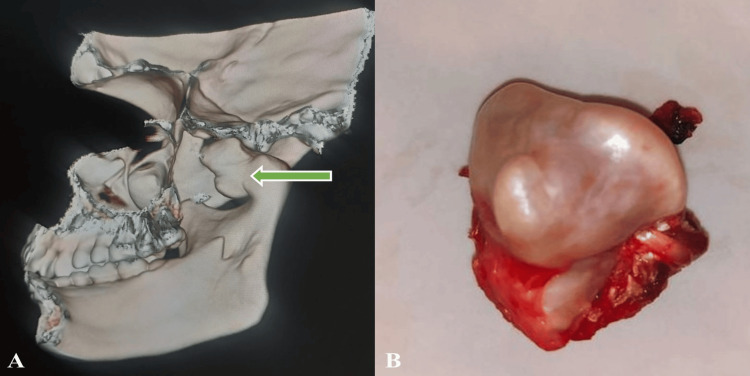
Descriptive images (A) 3D reconstruction of the involved mandibular condyle and (B) the excised specimen

Upon radiographic evaluation, a bony growth originating from the right condyle of the mandible, positioned in an anteromedial manner within the condyle itself, was noted. Computed tomographic 3D reconstruction revealed the presence of a large, lobulated dense osseous mass in continuity with the underlying bone, suggestive of an OC. Subsequently, a condylectomy procedure was carried out under general anesthesia (Figure 3). The excised specimen was fixed with 10% formaldehyde and submitted for histopathological examination. Upon gross macroscopic examination, bony hard tissue attached to soft tissue was seen. It was greyish-white in color, hard in consistency, and measured about 3.5 cm x 2 cm.

**Figure 4:**
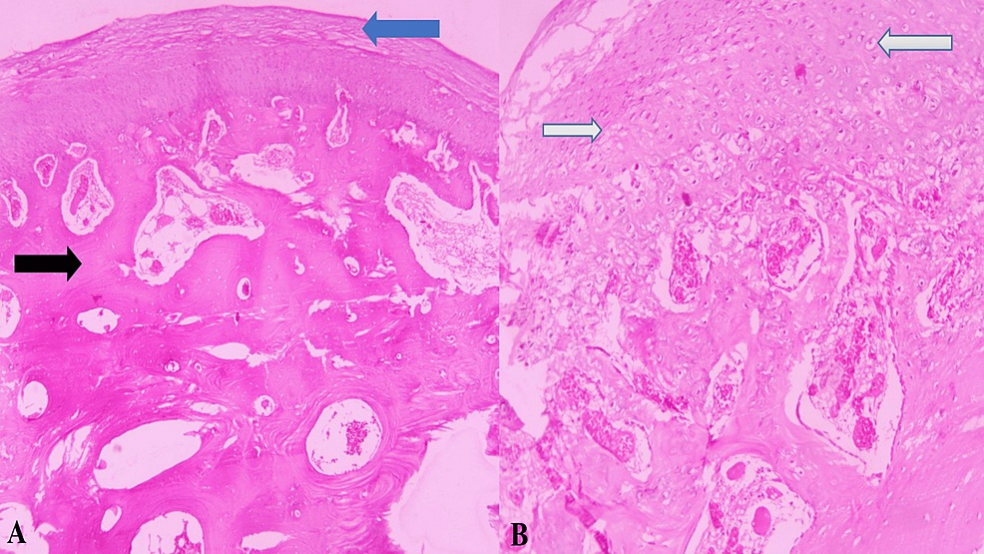
Photomicrographs Photomicrographs of hematoxylin and eosin stained sections in low power view showing fibrous perichondrium covering the cartilage cap (blue arrow) with periosteal continuity (black arrow) (A, H&E, 10x) and clusters of chondrocytes with small nuclei (white arrows with a blue outline) in the cartilaginous cap (B, H&E, 10x)

Histopathological examination by routine hematoxylin and eosin (H and E) staining showed a fibrous perichondrium covering the cartilage cap and in continuity with the periosteum of the underlying bone. At lower magnification, clusters of chondrocytes with small nuclei were discernible (Figure 4A). These chondrocytes were arranged in parallel within lacunar spaces within the cartilaginous cap. Toward the base of the cartilaginous cap, areas of endochondral ossification were apparent, which merged with the trabecular bone (Figure 4B). Within the intertrabecular spaces, hematopoietic marrow was also noted. Moderate vascularity, areas of hemorrhage, muscle tissue, and adipocytes were identified. As it was diagnosed with classic histopathological features, special stains and IHC were not performed.

Correlating the clinical, radiological, and histopathological findings, it was diagnosed as OC of the mandibular condyle. The patient remains disease-free on the eighth-month post-surgical follow-up.

## Discussion

In 2002, the World Health Organization (WHO) defined the OC as a bony projection covered by cartilage, originating from the external surface of a bone, and possessing a marrow cavity that is connected to that of the underlying bone [7]. This condition primarily emerges during the first to third decades of life, showing no significant gender preference, and typically involves the metaphysis of long bones [8].

Approximately 1% of OCs manifest in the head and neck region [6]. Within the head and neck area, the most prevalent locations are the coronoid process and the mandibular condyle [2]. Trauma and inflammation are considered predisposing factors for their development [2,9]. In terms of the tumor's location, around 52% of OCs occur on the medial aspect of the condyle, 20% are positioned anteriorly, and only 1% are found laterally on the condyle [10].

The OC of the mandibular condyle is typically accompanied by various signs and symptoms, encompassing facial asymmetry, malocclusion, cross-bite on the opposite side, lateral open-bite on the affected side, deviation during mouth opening, restricted movement, discomfort and clicking, and alterations in condylar morphology [6,10,11]. Our patient presented with mandibular deviation, altered bite, and restricted movement. Alterations in the condyle were observed during radiological investigations.

A hypothesis concerning the development of an OC within the condyle suggests the presence of anomalous foci of epiphyseal cartilage on the surface of the bone. Accordingly, the growth of this tumor could be attributed to stress within the insertion area of the lateral pterygoid muscle, wherein a concentration of cells with cartilaginous potential accumulates. This hypothesis gains support from the observation that the tumor most often emerges on the medial aspect of the condyle [6]. Hyperplasia of cartilaginous cells by tensional forces is the strongest hypothesis, because the condylar OC occurs commonly on the medial side (57%), followed by the anterior side (20%), and almost none occur in the lateral or superior aspects (<1%) [12].

When considering possible diagnoses, it is important to differentiate OCs from conditions such as condylar giant cell tumors, condylar hyperplasia, fibro-osseous lesions, vascular malformations, osteomas, chondromas, osteochondromatosis, and osteoblastomas [1,9,11]. Osteomas usually present as a pedunculated bony mass. Chondromas show irregular radiolucent mass with mature cartilage. Osteochondromatosis shows hot spots in areas other than the mandibular condyle on bone scans [12]. Fibro-osseous lesions, giant cell tumors, condylar hyperplasia, vascular malformations, and osteoblastomas could be ruled out directly with histopathological findings. Our case presented with classical histopathological findings of OCs.

Table 1 outlines the key differences between OCs, unilateral condylar hyperplasia, and osteomas in terms of their nature, origin, clinical presentation, radiographic appearance, histopathology, and treatment considerations [7,10] (Table 1).

**Table 1:** Comparative table

Characteristic	Osteochondroma (OC)	Unilateral Condylar Hyperplasia (UCH)	Osteomas
Nature	Benign cartilage and bone tumor	Excessive growth of condyle	Benign bone tumor
Origin	Develops from the growth plate. The metaphyseal region of the long bones is the most common site of involvement.	Arises from hyperactivity of condylar growth, The etiology of condylar hyperplasia includes trauma, partial hemihypertrophy, osteochondromatosis, and neurotrophic disturbances. Genetic, acquired, functional factors, and age groups, also have a role in morphological changes in condyle. The occurrence of condylar hyperplasia in siblings suggested that it could be genetic in origin, either autosomal dominant or Y-linked, although with only a few cases and two generations of history, it will be difficult to determine it with any degree of certainty.	Arises from osteoblasts
Growth Pattern	Projects away from the bone	Enlargement of condyle in the same direction	Nodule-like growth on bone surface
Clinical Symptoms	Facial asymmetry, malocclusion	Facial asymmetry, malocclusion, pain	Asymptomatic or local symptoms
Radiographic Features	Cartilage cap with bony stalk. Condyle shape is irregular and "Cauliflower/mushroom-like" appearance	An enlarged condyle with no stalk. The condyle shape is normal. Enlargement of condyle with distinct margins that could be identified with Scintigraphy	Well-defined uniformly opaque bony mass Expansion of condyle. Homogeneous density on CT
Histopathology	Cartilage cap and underlying bone	Hypertrophic condylar bone growth	Thick cortical lamellar bone
Treatment	Surgical removal if symptomatic	Surgical reduction if symptomatic	Surgical removal if symptomatic

Table 2 provides an overview of the diverse diagnostic methods available for radiographic diagnosis of the OC [2,9]. 

**Table 2:** Diagnostic modalities PET-CT: Positron emission tomography-computed tomography

Diagnostic Modality	Description
X-ray	Utilizes X-rays to visualize bone and cartilage structures.
CT Scan	Provides cross-sectional images for detailed assessment
MRI	Offers clear visualization of soft tissue and bone marrow involvement, also helpful in measuring the thickness of the cartilage cap.
Ultrasound	Employs sound waves to examine superficial structures and evaluate the thickness of the cartilage cap. Ultrasound helps to evaluate the cartilage cap thickness. The cartilage cap appears as a hypoechoic region situated above a hyperechoic bone [4].
Nuclear Imaging (Bone Scans using Tc 99m - Methyl Diphosphonate)	Involves injecting a radioactive tracer to highlight areas.
Bone Scintigraphy	Detects active bone growth and can assess multiple lesions in hereditary multiple exostoses. technetium-99m-labeled diphosphonates [ 99m Tc-MDP] scintigraphy, Thalium 201 scintigraphy is useful in identifying malignant change within benign lesions [4].
PET-CT	Useful for identifying malignant transformation of osteochondroma. Various fluorodeoxyglucose (FDG) spectrum uptake has been observed in primary and metastatic heterogeneous bone lesions. positron emission tomography (PET) and hybrid PET/computed tomography (PET/CT) systems have been focused on using 18 F-NaF for osseous imaging.
Angiography	Visualizes blood vessels using intravenous contrast agents. Can be coordinated with computed tomographic angiographic images of the joint
Arthroscopy	Directly examines joints and cartilage using a small fiberoptic endoscope.

Local recurrence for solitary OCs in instances involving long bones has been documented to be 2% [1,6,12]. Kwon et al. reported that the recurrence rate of mandibular condylar OC was 1.3% (3 out of 236 cases) [12].

Sun et al. have reported a 3D evaluation of the OC [13]. Saito et al. have reported a bilateral OC [14]. Zhou et al. have specified two growth patterns including an OC with stalk or an OC with a sessile base [8]. If it is a mild presentation, the condition can be evaluated through regular clinical monitoring and radiological assessments [8]. Depending on factors such as symptoms, duration, and the size of the lesion, surgical approaches for OCs vary. These can include either solely removing the tumor or performing condylectomy in combination with tumor excision, followed by reconstructive surgery [6,8,15].

The main treatment modality is condylectomy but it may result in lateral open bite on the contralateral side. Hence, simultaneous condylar reconstruction is required to maintain the ramus height and function of the TMJ. Reconstruction could be performed with grafts, distraction osteogenesis, and vertical ramus osteotomy [16]. Costochondral graft is routinely used for reconstruction but requires surgery at the second site. If artificial grafts are placed as replacements, additional surgery is required for graft retrieval. Distraction osteogenesis could restore ramus height and maintain occlusion by condylar reconstruction. Its drawback is poor long-term stability showing condylar asymmetry. Vertical ramus osteotomy avoids surgery on the second site and has reduced risks. Its shortcoming is that the mandibular contour is damaged on the treated side [16]. Mamatha et al. have reported that OCs can occur involving the mandibular angle, mandibular symphysis, and even in soft tissue without any connection to the mandible [17].

Some authors suggest that the condyle can be preserved as much as possible as osteochondroma has a recurrence rate of just 2% in solitary osteochondroma cases of long bones. Preserving the condyle has the advantage of preserving the vertical ramus height and stable occlusion which will eliminate the need for reconstruction [12]. We have summarized various therapeutic possibilities of OCs involving the mandibular condyle (Table 3).

**Table 3:** Treatment modalities

Treatment options for the management of Osteochondroma Involving the Mandibular Condyle		
Mild presentation - Regular clinical monitoring and radiological assessment		
Presentation with symptoms or functional difficulties - Surgical therapy is definitive and curative		
Types of surgical management	Reconstruction methods	Drawbacks
Condylectomy with tumor excision and without reconstruction	No reconstruction	Functional defects
Condylectomy with tumor excision and reconstruction	Reconstruction with autogenous grafts like Costochondral grafts, Sternoclavicular joints	Requires surgery at the second site
Condylectomy with tumor excision and reconstruction	Reconstruction with alloplastic grafts like high molecular weight polyethylene joints, close-fitting custom-made prosthesis	Requires second surgery for graft retrieval
Condylectomy with tumor excision and reconstruction	Reconstruction by distraction osteogenesis	Long-term stability is questionable
Condylectomy with tumor excision and reconstruction	Reconstruction by Vertical Ramus Osteotomy	Changes in mandibular contour
Conservative management by recontouring the involved condyle	Not needed	Risk of recurrence at the involved site.

The majority of the literature suggests that the OC has a better prognosis and minimal recurrence rate but with constant clinical/radiological follow-up [7,13,14,15,17].

Mahajan et al. have recommended frequent measurement of cartilage cap thickness to rule out recurrence [6]. Kwon et al. have also recommended further studies to identify the actual recurrence of the OC, as they have reported recurrence of the OC with conservative management [12].

Kishore et al. have recommended periodic measurement of mandibular length in younger patients to identify any discrepancies [18]. Friedrich et al. have reported negative immunohistochemical assessment with Insulin-like growth factor in a case of OC [19]. Gardner et al. have discussed various surgical options for OCs [20]. Chen et al. have classified OCs of the mandibular condyle based on the CT findings into Type-1 protruding expansion and Type-2 globular expansion [21]. Type-1 could be treated with local excision and Type-2 requires condylectomy.

There are EXT1 homozygous deletions in a solitary OC, whereas both EXT1 and EXT2 genes have been identified in multiple OCs [5]. Somatic mutations have been identified in chromosomes 8 and 11 [22].

In certain situations, there exists potential for malignant transformation, with low-grade chondrosarcomas being the most prevalent form of a malignant tumor to develop [1]. Malignant transformation is reported to be 1-2% in solitary OCs and 2-25% in osteochondromatosis [18]. de Souza et al. have reported that rapid size increase, continuous growth, local pain, or erythema in a previously asymptomatic OC raises suspicion of a malignant change [23]. Histopathological changes with architectural loss of cartilage, increased mitosis, cellular atypia, and necrosis indicate a malignant change.

HME is a rare genetic disorder and it is also called hereditary multiple osteochondromas, hereditary deforming dyschondroplasia, diaphyseal aclasis, and multiple cartilaginous exostoses [24]. Further studies on various biomarkers including heparan sulfate along with genetic analysis will help in understanding the pathogenesis of cartilage within this rare bone lesion.

## Conclusions

OCs constitute about 50% of benign bone tumors; however, their occurrence in the head and neck area is infrequent. Effective management entails thorough clinical, radiographic, and histopathological assessment. With a low recurrence rate of only 2%, the prognosis for this tumor is favorable following complete excision. We have presented such a rare presentation of an OC involving the left mandibular condyle of a young female along with its clinical, radiological, and histopathological findings.
